# Treatment of colorectal cancer by traditional Chinese medicine: prevention and treatment mechanisms

**DOI:** 10.3389/fphar.2024.1377592

**Published:** 2024-05-09

**Authors:** Jiaxin Sun, Ying Wei, Jia Wang, Mingxing Hou, Liya Su

**Affiliations:** ^1^ Affiliated Hospital of Inner Mongolia Medical University, Inner Mongolia Key Laboratory of Medical Cell Biology, Hohhot, Inner Mongolia, China; ^2^ Department of Gynaecology, Inner Mongolia People’s Hospital, Hohhot, Inner Mongolia, China; ^3^ Department of Gastrointestinal Surgery, Affiliated Hospital of Inner Mongolia Medical University, Hohhot, Inner Mongolia, China

**Keywords:** traditional Chinese medicine, colorectal cancer, prescriptions, herbs, components

## Abstract

Colorectal cancer (CRC) is a significant global health burden, with high morbidity and mortality rates. It is often diagnosed at middle to advanced stage, affecting approximately 35% of patients at the time of diagnosis. Currently, chemotherapy has been used to improve patient prognosis and increase overall survival. However, chemotherapy can also have cytotoxic effects and lead to adverse reactions, such as inhibiting bone marrow hematopoiesis, causing digestive dysfunction, hand-foot syndrome, and even life-threatening conditions. In response to these adverse effects, researchers have proposed using Traditional Chinese Medicine (TCM) as an option to treat cancer. TCM research focuses on prescriptions, herbs, and components, which form essential components of the current research in Chinese medicine. The study and implementation of TCM prescriptions and herbs demonstrate its distinctive holistic approach to therapy, characterized by applying multi-component and multi-target treatment. TMC components have advantages in developing new drugs as they consist of single ingredients, require smaller medication dosages, have a precise measure of pharmacodynamic effects, and have a clear mechanism of action compared to TCM prescriptions and herbs. However, further research is still needed to determine whether TMC components can fully substitute the therapeutic efficacy of TCM prescriptions. This paper presents a comprehensive analysis of the research advancements made in TCM prescriptions, herbs, and components. The findings of this study can serve as a theoretical basis for researchers who are interested in exploring the potential of TCM for the treatment of colorectal cancer.

## 1 Introduction

Colorectal cancer (CRC) is a prevalent form of cancer, ranking third in terms of occurrence and second in terms of mortality globally. In 2020, there were over 1.9 million new cases and 935,000 deaths, comprising roughly one-tenth of all cancer cases and fatalities (Sung et al., 2021). Notably, in China, the incidence and mortality of CRC are significantly increasing. Its incidence is expected to reach three million by 2024, making it one of the most menacing cancers in terms of lives and wellbeing (Qu et al., 2022; Morgan et al., 2023). CRC is a highly malignant disease characterized by quick disease progression and lymphatic and blood circulation metastasis. Advanced stages of CRC can lead to severe complications such as anemia and acute organ perforation. Thus, exploring efficacious remedies has become a focal point of research.

Currently, CRC treatment relies mainly on surgery, with additional therapies such as chemotherapy and targeted therapy. Surgical resection is a widely used approach for managing stage I and stage II colorectal cancer, demonstrating a promising 5-year survival rate of over 90% for stage I cases. However, the survival rate for advanced CRC is only 14% (Siegel et al., 2023). CRC is identified by its subtle early symptoms, with most patients not diagnosed until the intermediate or late stages of the disease, when symptoms appear and medical attention is sought. Medical advancements have enabled chemotherapy in combination with surgery to treat intermediate and late CRC patients, substantially improving primary tumor control and patient survival rates (Khalil et al., 2022). Chemotherapy has some benefits for patients but also brings various side effects, such as myelosuppression and infections due to impaired immune function. These side effects not only reduce patient compliance but also severely affect their quality of life, leading to the recurrence of tumor metastases and ultimately affecting patients’ long-term survival (Miller et al., 2019). As a result, finding an effective treatment for CRC becomes the focus of research hotspot at home and abroad.

**FIGURE 1:**
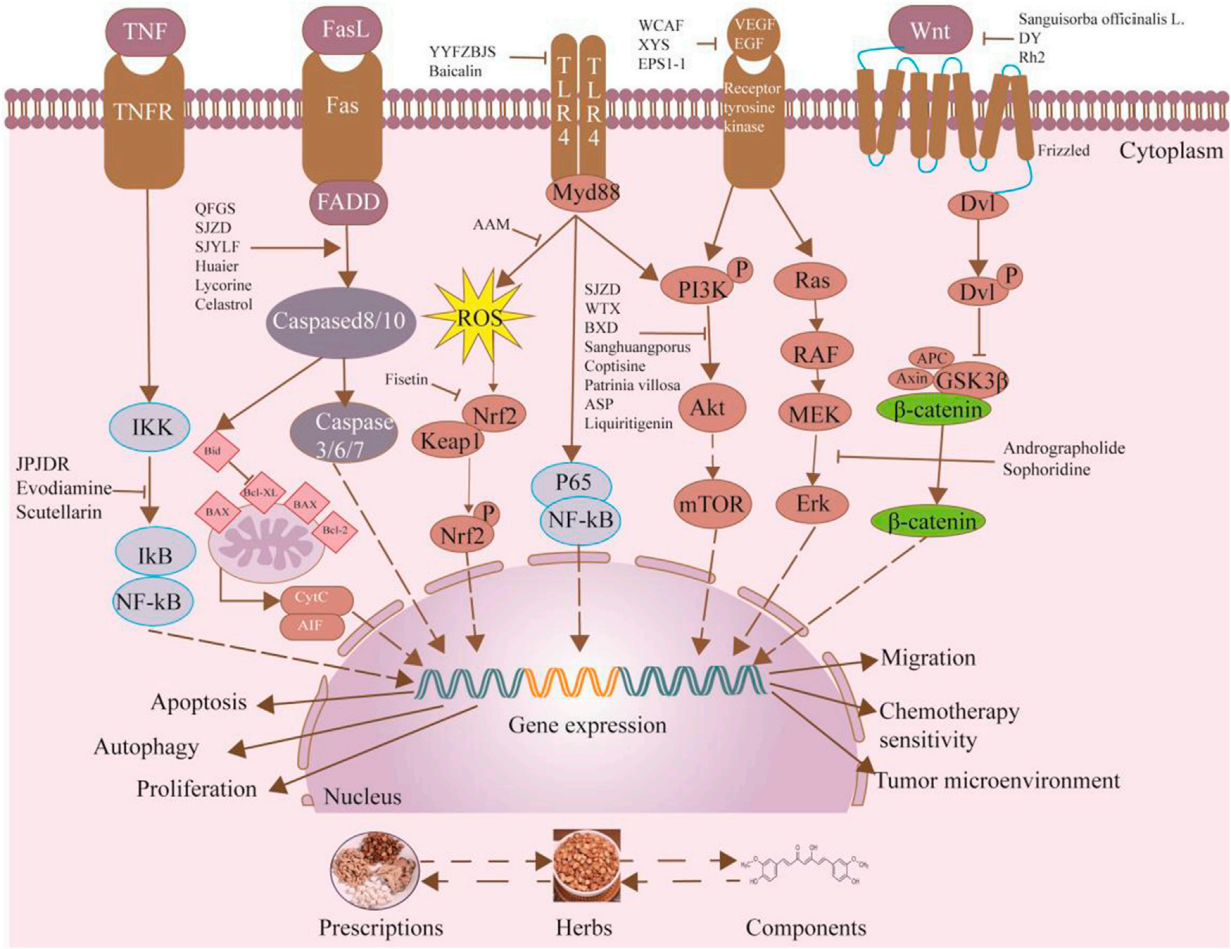
The parts of TCM contains prescriptions, herbs and components that participate in the treatment of CRC through different mechanisms.

Research has increasingly demonstrated that TCM have potent effects in treating cancer by experimental and clinical models. Therefore, they are being explored as therapeutic agents for CRC. TCM has been extensively researched and used for centuries. These medicines are primarily derived from botanical sources and are essential for open anticancer drugs (Kong et al., 2020). As a valuable treatment for CRC, TCM can have a multi-targeted impact on colorectal cancer, minimizing toxic side effects and extending patient survival periods caused by surgery, chemotherapy, radiotherapy, targeted therapy, and immunotherapy (Ranjan et al., 2019). Experimental research has demonstrated that TCM and its ingredients can efficiently impede the growth of CRC cells, trigger apoptosis, stimulate cell autophagy, and suppress angiogenesis; it also contributes to treat colorectal cancer when combined with radiotherapy (Chen J-F et al., 2023). TCM has a lengthy history and extensive clinical applications. TCM research usually focuses on prescriptions, herbs, and components (Sun et al., 2021). Prescriptions present notable benefits in inhibiting the proliferation and metastasis of (Wei et al., 2023). Herbs comprise a single medicinal ingredient and are extracted using various methods, resulting in increased CRC efficacy due to their high concentrations of active components and ability to reduce harmful side effects (Xia et al., 2014). Compared with prescriptions and herbal medicines, ingredients have clear chemical structures and pharmacological functions and have become an important part of the research and development of new TCM treatments (Guo T-H et al., 2022). This paper will review the research advancements of prescriptions, herbs, and components in CRC. We will explore their mechanisms of action, avoid subjective evaluations, and provide a theoretical basis for future research on TCM’s effectiveness against CRC (Figure 1).

## 2 Application of prescriptions in CRC

The study and implementation of prescriptions in TCM demonstrate its distinctive holistic approach to therapy, characterized by applying multi-component and multi-target. Similar to the compound presented in this paper, it is categorized based on its primary effects, including inhibition of apoptosis and proliferation, the inhibition of migration, invasion and adhesion, the regulation of gut microbiota and other mechanisms (Table 1).

**TABLE 1:** Lists of TCM prescriptions with potential anti-CAC action.

Regulatory mechanism	TCM formula	Composition	Model	Optimal dose	Effects and potential mechanism	Ref
Inducing apoptosis and inhibiting proliferation	Qingjie Fuzheng granules (QFGs)	Scutellaria barbata D. Don, malt, Hedyotis diffusa Willd, and Astragalus	HCT-8 and HCT-116	0.5, 1, and 2 mg/mL	Increased the expression levels of Bax, Fas and FasL, decreasing the level of Bcl-2, and stimulating the activation of caspase-3/-8/-9, inducing apoptosis in CRC cells	Yang et al. (2019)
	Sanjie Yiliu formula (SJYLF)	Rhizoma Pinelliae Preparatum (Fabanxia), Glabrous Sarcandra herb, Thunberg Fritillary bulb, and ground beetle species	HCT-8, SW-480 HT-29, and DLD-1	0.5,1.0, 1.5 mg/mL	Suppressed proliferation and inducing apoptosis in CAC cells and downregulating cyclin D1, CDK4, and BCL-2, while Bax expression was upregulated	Tang et al. (2021)
	Sijunzi Decoction (SJZD)	Panax ginseng, Atractylodes macrocephal, Poria cocos and Glycyrrhiza uralensis	HCT116 LOVO	50, 75, and 100 µg/mL	Promoted apoptosis and autophagy of CRC cells through PI3K/Akt/mTOR pathway	Dong S et al. (2022)
			thymic BALB/C nude mice	3.5, 7, and 14 g/kg		
	Wei-Tong-Xin (WTX)	Rheum offcinale Baill., Pharbitis nil (L.) Choisy. (PNC), Aucklandia lappa Decne., Gleditsia sinensis Lam. (GSL), Glycyrrhiza uralensis Fisch. (GUF)	HCT116	50, 100 µg/ml	Induced of apoptosis via PI3K/AKT signaling pathway	Lin et al. (2023)
			BALB/c mice	2.8,1.4, 0.7 g/kg		
	Yi-Yi-Fu-Zi-Bai-Jiang-San (YYFZBJS)	Coicis Semen, Aconiti Lateralis Radix Praeparata, and Herba Patriniae.	HT29	20 µg/mL	Downregulated SMOX expression via anti-inflammatory signaling and regulation of the TLR4/NF-κB signaling pathway.	Li J et al. (2022)
			BALB/c nu/nu mice	50 mg/kg		
	Weichang’an formula (WCAF)	Taizishen, Baishu, Daxueteng, Tengligen planch.ex Miq., Baqia, Chenpi, Xiakucao	Nude mice	0.368 g/mL	Induced CRC apoptosis and decreased the expression of Leptin, VEGF-A and VEGFR-1	Pan et al. (2020)
Inhibiting metastasis	Xiaoyaosan (XYS)	Chinese Thorowax Root, Radix Angelicae Sinensis, Radix Paeoniae Alba, Rhizoma Atractylodis Macrocephalae, Poria, Radix Glycyrrhizae, Herba Menthae, Rhizoma Zingiberis Recens	BALB/C nu/nu mice	1516.67 mg/kg	Reduced VEGF and CD31 in hepatic metastatic tissue and inhibit chronic stress induced liver metastasis	Zhao et al. (2020)
	JianPi JieDu Recipe (JPJDR)	Astragalus membranaceusceus, Panax quinquefolius, Atractylodes macrocephala, Poria cocos, Coix seed, Smilax china, Hedyotis diffusa, Sculellaria barbata, Paris polyphylla, Actinidia argut, and Glycyrrhiza uralensis Fisch	LoVo, HCT116, MC-38, LX-2	50, 100, 200 µg/mL	Reduced CRC liver metastasis by regulating ITGBL1-rich EVs secretion from CRC and blocking the fibroblasts activation by regulating ITGBL1-TNFAIP3- NF-κB signaling.	Li R et al. (2022)
			C57BL/6 mice	50, 100, 200 µg/mL		
	Astragalus Atractylodes mixture (AAM),	Astragalus membranaceus Fisch. ex Bunge. (AMF), Atractylodes macrocephala Koidz. (AMK), Actinidia arguta (Siebold & Zucc.) Planch. ex Miq. (AAP), Curcuma aromatica Salisb. (CAS), Benincasa hispida (Thunb.) Cogn. (BHC), and Ficus pumila L. (FPL)	HCT-116, LoVo	2.5, 5, 10 mg/mL	Inhibited migration and VM formation by suppressing ROS/HIF-1a/MMP2 pathway in colorectal cancer	Zong et al. (2020)
			athymic nude mice	16 mg/g		
	Sini decoction (SND)	Fuzi, Zhigancao, Ganjuang,	BALB/c male mice	189 mg/500 mL	Limited CRC liver metastasis and upregulated IL-2 and IFN-γ	Chen J et al. (2023)
	Jianpi Jiedu Recipe	astragalus, Poria, atractylodes, three li, zedoary, Dangshen, sand ren	C57BL/6 mice	10 µg/100 µL	Inhibited colorectal cancer metastasis by suppressing the extracellular vesicle-mediated expression of ITGBL1	Li R et al. (2022)
	Chanling Gao(CLG)	Grass black, wood turtle kernel, spirit fairy, phoenix fairy, toad venom	male BALB/c nude mice	Dosage unknown	Limited CRC metastasis and reduced MMP-2 and MMP-9 expression in tumors	Chen et al. (2024)
Regulating the intestinal microbiota	Xiaoai Jiedu recipe (XJR)	Hedyotis diffusa, Radix pseudostellariae, Akebiatrifoliata Koidz, Radix ophiopogonis, Bombyx batryticatus, Cremastra appendiculata, and Centipede	DLD-1 cells	drug serum	Bacteroidetes, *Bacteroides*, and Prevotellaceae decreased, but the levels of beneficial bacteria increased (Firmicutes, Roseburia, and Actinobacteria)	Qiu et al. (2023)
			BALB/c mice	5, 20 g/kg		
	Pien-Tze-Huang (PZH)	musk, Calculus bovis, snake gall, and Panax notoginseng roots	AOM/DSS, Apcmin/+ mice	270 and 540 mg/kg	Inhibited colorectal tumorigenesis in AOM/DSS treated mice and in Apcmin/+ mice in a dose-dependent manner. PZH treatment altered the gut microbiota profile	Gou et al., 2023
	Yi-Yi-Fu-Zi-Bai-Jiang-San (YYFZBJS)	Coicis Semen, Aconiti Lateralis Radix Praeparata, and Herba Patriniae.	C57BL/6 J ApcMin/+ mice	3.825, 7.65, 15.3 g/kg	Elevated probiotic genera (Bifidobacterium and prevotellaceae) and reducing *bacteroides*, Lachnospiraceae, *Lactobacillus* and Dubosiella.	Sui et al. (2020)
	Wu Mei Wan (WMW)	Fructus Mume, Rhizoma Coptidis, Herba Asari Mandshurici, Ramulus Cinnamomi, Radix Ginseng, Radix Aconiti Lateralis Preparata, Pericarpium Zanthoxyli Bungeani, Rhizoma Zingiberis, Cortex Phellodendri Amurensis, and Radix Angelicae Sinensis	C57BL/6 mice	5.8 g/kg/d	Bacteroidetes decreased (*p* < 0.05) and Firmicutes increased, At the family level, compared to the NC group, the bacteroidales_s24-7_group (*p* < 0.01) and Lachnospiraceae significantly decreased.	Jiang et al. (2020)
Other mechanisms	DangguiBuxue Tang (DBT)	Astragali Radix (AR) and Angelicae Sinensis Radix (ASR)	CT26 and HT-29	2.98 mg/mL, 10.1 mg/mL	Induced autophagy-associated cell death of CT26, sensitized to chemotherapy and radiotherapy treatment and inhibited the growth of CRC	Chen et al. (2016)
	T33	Kansui Radix, Glycyrrhizae Radix et Rhizoma Praeparata cum Melle, Paeoniae Radix Alba, Pinelliae Rhizoma Praeparatum Cum Zingibere et Alumine, and Rhei Radix et Rhizoma	HT-29 and Caco2	0, 0.1, 0.5, 2.5, 5, 10 mg/mL	Inhibited CRC activity by promoting autophagy and Increased Atg7, Atg5, and Beclin-1 proteins	Liu et al. (2022)
	Banxia Xiexin decoction (BXD)	Pinellia, Scutellaria, dried ginger, ginseng, grilled licorice, Coptis, jujube	HCT116 and SW480	150, 304, and 600 μg/mL	Increased the ratio of LC3 II/LC3 I and NCOA4, and reduced the levels of FTH1 and GPX4 through suppression of the PI3K/AKT/mTOR axis	Y. Wang et al., 2024
	Huoxiang Zhengqi (HXZQ)	Rhizoma Atractylodis, Citrus reticulata, Cortex Magnoliae officilis, Radix Angelicae Dahuricae, Poria, Pericarpium ArecaeAreca, Rhizoma Pinelliae, Radix Glycyrrhizae, Oleum Pogostemonis, Oleum Folii Perillae	AOM/DSS	0.45 or 1.35 g/kg	Activated Nrf2 signaling pathway and increased the levels of antioxidants, suppressing the size and number of tumors.	Dong M et al. (2022)
	Shaoyao decoction (SYD)	Scutellaria baicalensis, Coptis chinensis, Paeonia lactiflora, Angelica sinensis, Mucuna pruriens, Betel nut, Rhubarb, Cinnamon, Radix et Rhizoma Glycyrrhizae	HT29	2,4,6,8 mg/mL	Activated Nrf2 pathway and upregulating expression of Nrf2 downstream genes, exerting anti-inflammatory and anti-oxidant effect in AOM-induced murine model of colon cancer	Wang X et al. (2020)
	Shenling Baizhu Decoction (SLBZD)	Ginseng, White Atractylodes, Poria, Licorice, Coix Seed, Amomum, Hyacinth Bean, Chinese Yam, Balloon Flower Root, Lotus Seed	BALB/c-Hpd1 mice	0.5 g/mL	Increased M1 macrophages and decreased M2 macrophages and Treg cells in the tumor immune microenvironment	Deng et al. (2024)
	Bazhen Decoction (BZD)	Ginseng, Atractylodes macrocephala, Poria cocos, Angelica sinensis, Chuanxiong, Paeonia lactiflora, Rehmannia glutinosa, and licorice	HCT116, SW620, and MC38	0, 1, 2, 4 mg/mL	Treated CRC through regulating tumor immune microenvironment	Lu et al. (2023)
			Female C57BL/6 mice	6.63 g/kg		

### 2.1 Inducing apoptosis and inhibiting proliferation

Traditional Chinese medicine Qingjie Fuzheng granules (QFGs), consisting of malt, Scutellaria barbata D. Don, Hedyotis diffusa Willd, and Astragulus, showed the function of inhibiting proliferation and inducing apoptosis at concentrations of 0.5–2.0 mg/mL. QFGs increased the expression level of Bax, Fas, and Fasl, decreased Bcl-2 levels, and stimulated activation of caspase-3/8/9 in HCT-116 and HCT-8 cell. This study showed that QFGs induced apoptosis via the mitochondria-dependent pathway and the death receptor apoptosis pathway in two types of CRC cells (Yang et al., 2019). Tang et al. conducted an experiment that differed from the Qingjie Fuzheng granule study. The experiment selected four CRC cells and found that the Sanjie Yiliu formula (SJYLF) significantly inhibited the activity of HCT-8, SW480, HT29, and DLD-1 CRC cells. SJYLF components, consisting of Fabanxia, Glabrous Sarcandra herb, Thunberg Fritillary bulb, and ground beetle species, initiated apoptosis through downregulating Bcl-2, cylin D1, and CDK4 protein, as well as increasing Bax expression (Tang et al., 2021). Besides, Sijunzi decoction (SJZD) consistsing of Panax ginseng C.A.Mey., Atractylodes macrocephala Koidz., Poria cocos Wolf., and Glycyrrhiza uralensis Fisch., proportioned at a ratio of 2:2:2:1 and clinically used in treating CRC. Experimentally, SJZD could induce apoptosis and autophagy of CRC cells via PI3K/Akt/mTOR pathway. This article analyzed the function of SJZD through network pharmacology technology and experimental *in vivo* and vitro (Shang et al., 2023). The PI3k/AKT pathway can regulate the proliferation and cycle of tumor cells, promote tumor angiogenesis, facilitate tumor invasion and metastasis, and regulate apoptosis (Dong S et al., 2022). Zhang and others, being similar to the previous of SJZD experiment, discovered 286 bioactive compounds and 130 potential therapeutic targets in the ethanolic extract of gastric tonic. They demonstrated that Wei-Tong-Xin induces colon cancer cell apoptosis by activating the PI3K/AKT pathway instead of iron death in HCT116 cells. The western blot analysis revealed increased expression of Bax, caspase 3, and caspase 9, and decreased expression of BCL-2. Additionally, Zhang and others conducted *in vivo* mouse experiments to validate the apoptotic role of Wei-Tong-Xin in colorectal cancer. They also confirmed the apoptotic effect of Wei-Tong-Xin on colorectal cancer through *in vivo* mouse experiments (Lin et al., 2023). Contrast to the previous western blot analysis, Weichang’an formula (WCAF) plays an important role in inducing CRC apoptosis through TUNEL assay. And the treatment group of WCAF decreases the expression of Leptin, VEGF-A and VEGFR-1 (Pan et al., 2020).

Yi-Yi-Fu-Zi-Bai-Jiang-San (YYFZBJS) comprises Coix lacryma, Radix et Rhizoma Pinelliae and Radix et Rhizoma Bianchi, three Chinese herbs combined in a 30:6:15 ratio. It is commonly administered for the treatment of gastrointestinal tumours. YYFZBJS, at concentrations of 30 µg/mL, 60 µg/mL, and 90 µg/mL, significantly decreased the expression of CDK1, p-AKT, and p-PI3K proteins in HCT116 and SW480 cells. This finding proves that by regulating the CDK1/PI3K/AKT pathway, inducing apoptosis and blocking the cell cycle, YYFZBJS effectively inhibits the proliferation of CRC. The efficacy of YYFZBJS on proliferation was validated by establishing the AOM/DSS mouse model (Li J et al., 2022). In an identical study, Xiang et al. conducted a network pharmacological analysis to screen four active ingredients from the YYFZBJS recipe. These four active ingredients were identified using high-performance liquid chromatography and were included in the HT-29 cell culture medium. The study found that the four active ingredients efficiently inhibited the growth and induced apoptosis of CRC cells by regulating TLR4/NFBJS (Xiang et al., 2022).

### 2.2 Inhibiting migration, invasion and adhesion

Metastatic cases of CRC can be detected at initial diagnosis in 20%–25% of CRC patients (Piawah and Venook, 2019). CRC patients drops to 14% after metastasis for 5-year survival rate (Siegel et al., 2023). Metastasis of tumors poses a significant challenge in the current treatment of CRC. Studies have confirmed that angiogenesis is a key factor in tumor metastasis, and vascular endothelial growth factor (VEGF) plays a crucial role in angiogenesis (Sakata and Larson, 2022). Lu Zhao et al. administered a safe clinical Chinese medicine, Xiaoyaosan, to C57BL/6J mice in an aqueous solution of 1516.67 mg/kg through gavage for 7 days. HT-29 colon cancer cells were then injected into the spleen of mice in order to establish a liver metastasis model of C57BL/6J colon cancer, and they continued to receive the treatment for 21 days. The study found that the Xiaoyaosan group had a significant inhibitory effect on liver metastasis of colon cancer by reducing the expression of VEGR and CD31 in liver metastatic tissues (Zhao et al., 2020). In addition, tumor cells can interact with the extracellular matrix, creating a pipeline system that transports blood, also known as angiogenic mimicry. This process leads to remodeling of the tumor microenvironment and is related to metastasis and prognosis (Wang et al., 2017). Zong et al. established a nude mouse model of lung metastasis by administering an Astragalus Atractylodes mixture, 16 mg/g, by gavage for 50 days. Zong found that the number of instances of lung metastasis in the Astragalus Atractylodes mixture group was significantly lower. The result showed that the mixture of Astragalus Atractylodes effectively inhibited CRC angiogenesis mimicry and migration of HCT116 and LOVO cells (Zong et al., 2020). Sini decoction (SND) consists of Fuzi, Zhigancao and Ganjuang, limiting CRC liver metastasis and upregulating IL-2 and IFN-γ. The effective of SND is associated with PI3K-Akt, EGFR and HIF-1 signaling pathway (Chen J et al., 2023).

The Jianpi Jiedu Recipe is a traditional Chinese medicinal compound derived from clinical practice that has found extensive use in treating gastrointestinal tumors. C57BL/6 mice were injected with 10 µg/100 µL Jianpi Jiedu Recipe into the tail vein every other day for 3 weeks, followed by an intra-splenic injection of MC38 colorectal cancer cells. The study revealed that Jianpi Jiedu Recipe effectively inhibits colorectal cancer metastasis by suppressing the extracellular vesicle-mediated expression of ITGBL1, inhibiting the TNFAIP3-NF-kB pathway activity, and subsequently reducing the activation of CAFs (Li R et al., 2022). Compared to regulate TNFAIP3-NF-kB pathway activity, a novel research shows that chanling Gao (CLG), a Chinese medicine formula, can limit CRC metastasis and reduce MMP-2 and MMP-9 expression in tumors. The result indicate that CLG regulate the PI3K/Akt/mTOR signaling pathway to inhibit metastasis of CRC (Chen et al., 2024).

### 2.3 Regulating the gut microbiota

The gut microbiota is a highly complex system that regulates innate and adaptive immunity. Disruption of gut microbiota can result in the procession of colorectal cancer (Jain et al., 2021). Patients with CRC often have dominant gut microbiota consisting of certain germs such as *Escherichia coli*, *Bacillus* fragilis, and *Clostridium* nucleatum. These pathogenic bacteria interfere with the immune surveillance mechanism by impairing intestinal mucosal immunity, promoting CRC development (Clay et al., 2022). Pien-Tze-Huang (PZH) can deplete pathogenic bacteria Peptoniphilus harei, *Campylobacter* jejuni, Collinsella aerofaciens and *Aeromonas* veronii in AOM/DSS mice and Apcmin/+ mice. At the same time, PZH inhibited tumorigenesis of CRC through increasing the abundance of probiotics Eubacterium limosum and Pseudobutyrivibrio xylanivorans (Gou et al., 2023).

Xiaoai Jiedu Recipe (XJR) is a kind of traditional Chinese medicine prescription for the treatment of colorectal cancer. 5 g/kg and 20 g/kg of XJR was used to treat CRC in xenograft model of mice by gavage for 14 consecutive days. Using 16s rRNA gene sequencing, the XJR dosing group decreased the abundance of Prevotellaceae, *Bacteroides* and Bacteroidetes. Studies demonstrated that XJR can inhibit the development of CRC in mice by modulating gut microbiota (Qiu et al., 2023).

Compared to the xenograft model of mice, Sui Hua et al. chose C57BL/6J-APCmin/+ mice to investigate the role of Yi-Yi-Fu-Zi-Bai-Jiang-San (YYFZBJS). This study used healthy controls and feces from volunteers receiving YYFZBJS to gavage APCmin/+ mice for 12 weeks. Contrasted to the healthy control group, mice receiving feces from volunteers receiving the drug had a reduced number of intestinal tumors, and gut microbiota was significantly regulated, as evidenced by an increase in the fractionation of Bifidobacterium and Prevotellaceae and a decrease in the abundance of *Bacteroides*, Lachnospiraceae, and Dubosiella. The altered gut microbiota mediated by YYFZBJS repressed CRC cell growth (Sui et al., 2020).

Feng et al. chose the AOM/DSS mouse model to conduct their study to explore Wu Mei Wan’s mechanism. Wu Mei Wan (WMW) was derived from the Treatise on Typhoid Fever and can treat abdominal pain and dysentery. The results indicated that after WMW intervention, the abundance of Bacteroidetes decreased, and that of Firmicutes increased at the phylum level. Additionally, the abundance of Bacteroidales_s24-7_group decreased, while that of Lachnospiraceae increased at the family level. WMW regulated NF-kB/IL-6/STAT3 pathway to balance between tumor-promoting and tumour-suppressing bacteria, thereby attenuating CAC (Jiang et al., 2020). Contrast to NF-kB/IL-6/STAT3 pathway, Anchang Yuyang Decoction (AYD) can regulate PPAR signaling pathway in CRC. AYD treatment group showed that the relative abundance of genera decreaesed, including Romboutsia, Monoglobus, norank_f_Oscillospiraceae, norank_f_ruminococcaceae, and other generas upregulated, such as norank_f_Muribaculaceae, *Bacteroides*, unclassified_f_Prevotellaceae, and Alistipes (Wei et al., 2024). Above all, there is a great importance to regulate the intestinal flora. The balance of intestinal flora is related to CRC. The application of prescriptions in CRC has benefit on the balance of intestinal flora, so that it can effectively treat CRC.

### 2.4 Other mechanisms

Autophagy is a free cellular mechanism of action to maintain homeostasis in response to various external stimuli, and in the case of tumours, excessive autophagy leads to autophagic cell death by degrading the cytoplasm beyond recovery (Mariño et al., 2014). The polysaccharide-depleted fraction of DangguiBuxue Tang (DBT) induced autophagy-associated cell death of CT26, sensitizing to chemotherapy and radiotherapy treatment and inhibiting the growth of CRC (Chen et al., 2016). What’s more, T33 is composed of five traditional Chinese herbs, namely, Kansui Radix, Glycyrrhizae Radix et Rhizoma Praeparata cum Melle, Paeoniae Radix Alba, Pinelliae Rhizoma Praeparatum Cum Zingibere et Alumine, and Rhei Radix et Rhizoma. T33 inhibits CRC activity by promoting autophagy, increasing Atg7, Atg5, and Beclin-1 proteins in HT-29 and Caco2 cells (Liu et al., 2022). Banxia Xiexin decoction (BXD) promoted ferritinophagy in CRC cells. BXD increased the ratio of LC3 II/LC3 I and NCOA4, and reduced the levels of FTH1 and GPX4 through suppression of the PI3K/AKT/mTOR axis (Wang et al., 2024).

Besides, inflammation and oxidative stress-induced carcinogenesis play significant part in the progression of CRC (Balmus et al., 2016). Jianpi Yiqi decoction is a commonly used treatment for gastrointestinal ailments like gastritis and colitis. Research found a significant decrease in IL-6 and TNF-a in venous blood, indicating that Jianpi Yiqi decoction has excellent anti-inflammatory properties and significantly reduces inflammatory responses. This result shows that good clinical efficacy was reflected in treating CRC patients through using Jianpi Yiqi prescription (Yang et al., 2023). It’s related to inflammatory in the next research which includes Huoxiang Zhengqi (HXZQ) significantly to reduce inflammation and oxidative stress in colitis-associated cancer by regulating Nrf2/NF-kB/NLRP3 pathway (Dong M et al., 2022). Compared with the study of HXZQ, ShaoYao decoction (SYD) also can activate the Nrf2 pathway, upregulating the expression of downstream Nrf2 genes and attenuating oxidative stress in AOM/DSS model mice. SYD can prevent and treat the ulcerrelated colorectal cancer (Wang X et al., 2020).

Additionally, we can’t aviod to mention the importance of the tumor microenvironment. A lot of researches found tirelizumab (TLzmab) resulted in imbalance of tumor immune microenvironment during treating CRC. Shenling Baizhu Decoction (SLBZD) can increase M1 macrophages and decrease M2 macrophages and Treg cells in the tumor immune microenvironment. Thus SLBZD has exerted the synergistic effect of TLzmab for maintaining the balance of microenvironment (Deng et al., 2024). Compared to regulate the macrophages in the microenvironment, Bazhen Decoction (BZD) can increase the ratio of CD4+T cells to CD8+T cells in the spleen and tumor tissues, downregulate the PD-1 expression on T cell surfaces. The study indicated BZD treated CRC through regulating tumor immune microenvironment (Lu et al., 2023). Thus, the prescriptions of TCM can also treat CRC through diversity mechanisms, which are potential targets to explore.

## 3 Application of herbs in CRC

In recent years, numerous herbs and extracts have demonstrated remarkable therapeutic effects in treating CRC (Xia et al., 2014). As the development of TCM, natural products have been widely applied in the treatment cancer. Natural products included traditional and herb medicines, abundant of researches will reveal their biofunctions and applications in cancer therapy (Newman and Cragg, 2016). These are commonly categorized by their extraction solution, including aqueous extract, ethanol extracts, and ethyl acetate extracts in traditional and herb medicines. Notably, varying extraction techniques of the same herb yield different pharmaceutical activity.

### 3.1 Inducing apoptosis and inhibiting proliferation

The trametes robiniophila murr (Huaier) were extracted with 95% anhydrous ethanol. Huaier extract improved the severity of tumorigenesis of CRC, reducing tumor number, size and load. After using Huaier, the apoptosis-associated protein levels, such as P53, Bax, and Bcl-2, showed significant differences. The results demonstrated that huaier extract suppressed cell proliferation and induced apoptosis in HCT116 and HCT8 cells (Zou et al., 2020). Compared with 95% ethanol and water extracts, the 60% ethanol extract of Sanghuangporus vaninii significantly inhibited the AKT/mTOR signaling pathway, as well as induced cell apoptosis and blocked G2/M cell cycle (Guo S et al., 2022). Patrinia scabiosaefolia also regulates the AKT pathway. The ethanol extract of Patrinia scabiosaefolia significantly reduced HCT-8/5-FU cell number and apoptosis (Huang et al., 2019). Additionally, Sanguisorba officinalis L. (DY) was extracted by aqueous. The aqueous extract of DY can suppress cell proliferation and apoptosis via increasing the expression of Bax, cleaved-caspase3 and cleaved-PARP proteins and reducing Bcl-2 expression (Zhang W et al., 2022). Salvia miltiorrhiza belongs to the Salvia genus. Salvia miltiorrhiza was dissolved in 100% dimethyl sulfoxide (DMSO). This study was based on network pharmacology and molecular docking technology, showing that Salvia miltiorrhiza was related to three key targets: SRC, IL-6, and INS. *In vitro* experiments, Salvia miltiorrhiza inhibited the proliferation of CRC via inhibiting the INS/SRC/IL-6 pathway (Jiang and Xun, 2024).

What’s more, some researchers had different view about using herbs to treat CRC. Patrinia villosa Juss. (P.V) can reduce the levels of CRC biomarkers CEA, CA19-9 and CA72-4 via PI3K/Akt signaling pathway (Li X-C et al., 2023). Besides, many studies though that a hig-fat diet (HFD) accelerates the risk of CRC. Jin found that Aster glehni (AG) had anti-adipogenic effects in mice model. AG inhibited colitis-associated colon carcinogenesis in mice via preventing colon shortening and reducing the number of colorectal polyps though inhibiting STAT3 (Jin et al., 2020). Above all, numerous herbs and extracts of TCM have remarkable therapeutic effects in inducing apoptosis and inhibiting proliferation of CRC.

### 3.2 Inhibiting migration, invasion and adhesion

The hot water extract of Melissa officinalis (MO) has more effective anti-CRC activity. By modulating the epithelial-mesenchymal transition (EMT), MO can inhibit migration, proliferation, and trigger apoptosis of CRC (Kuo et al., 2020). Compared with the hot water extract, the ethyl acetate extract 3 (EA3) of Bolbostemma paniculatum (Maxim.) Franquet can effectively suppress the clone formation, invasion and migration of CRC by suppressing the PI3K/Akt pathway (Li Y et al., 2022). Besides, Asparagus (ASP) can regulate the PI3K/AKT/mTOR signaling pathway, inhibiting proliferation, invasion and migration of HCT116 and LOVO cells (Liang et al., 2022). A similar result showed that Scutellaria barbata D.Don (SB) can effectively inhibit the migration and invasion ability of HCT-8 cells in a dose-dependent manner via PI3K/Akt and TGF-β pathways. The ethanol extract of SB can reduce the expression of MMP-1, MMP2, MMP-3/10, MMP-9, and MMP-13. And E-cadherin and N-cadherin had no significantly difference in using the ethanol extract of SB (Jin et al., 2017). Beside, Sanguisorba officinalis Linn. (DY) can reverse EMT procession, so that inhibition cell metastasis. After DY treatment, the results showed that DY can reduce the expression of N-cadherin, vimentin and snail proteins, and upregulate E-cadherin expression via inhibition of the Wnt pathway (Zhang W et al., 2022).

Furthermore, when Angelica sinensis and OXA act in combination on HCT116 cells, the combinations show synergistic or additive effects. The expression levels of Ki67, MMP9, and CD206 in the Angelica sinensis group combined with OXA group were lower than those in the OXA group. The results suggest that Angelica sinensis can be used as an auxiliary drug in the treatment of colorectal cancer (Hao et al., 2022). More detailed information concerning anti-CRC of herbs is depicted in Table 2.

**TABLE 2:** Lists of Herbs with potential anti-CAC action.

Regulatory mechanism	Herbs	Extraction solution	Model	Dosage	Effects and potential mechanism	Ref
Inducing apoptosis and inhibiting proliferation	Huaier	95% anhydrous ethanol	AOM/DSS Mode	4 g/kg	Suppressed cell proliferation and induced apoptosis in HCT116 and HCT8 cells	Zou et al. (2020)
			HCT116 and HCT8	4 mg/mL, 8 mg/mL		
	Sanghuangporus vaninii	60% ethanol extract	SW480	7.91 µg/mL	Inhibited the AKT/mTOR signaling pathway, as well as induced cell apoptosis and blocked G2/M cell cycle	Guo S et al. (2022)
	Patrinia scabiosaefolia	Ethanol extract	HCT-8/5-FU cells	0, 0.5, 1 or 2 mg/mL	Suppressed of the AKT pathway and promoted of cancer cell apoptosis	Huang et al. (2019)
	Sanguisorba officinalis L.	Aqueous extract	RKO-P/R and HCT15-P/R cells	124.2 and 105.2 μg/mL	Suppressed the growth and metastasis of 5-FU-sensitive and -resistant CRC via inhibition of the Wnt pathway	Yi et al. (2022)
	Salvia miltiorrhiza	100% dimethyl sulfoxide (DMSO)	HCT116 and DLD-1	16.89 μg/m, 16.89 μg/m	Inhibited the proliferation of CRC via inhibiting the INS/SRC/IL-6 pathway	Jiang and Xun (2024)
	Patrinia villosa	70% ethanol	AOM/DSS Mode	3.51 mg/20 g, 1.17 mg/20 g, 0.39 mg/20 g	Reduced the levels of CRC biomarkers CEA, CA19-9 and CA72-4 via PI3K/Akt signaling pathway	Li X-C et al. (2023)
	Aster glehni	70% ethanol	AOM/DSS Mode	25 and 50 mg/kg	Prevented colon shortening and reduced the number of colorectal polyps though inhibiting STAT3	Jin et al. (2020)
Inhibiting migration, invasion and adhesion	Melissa officinalis (MO)	Aqueous extract	HCT116	250 µg/mL, 375 µg/mL	Reduced cell proliferation and induced cell cycle arrest at the G2/M phase	Kuo et al. (2020)
	Bolbostemma paniculatum (Maxim.) Franquet	Ethyl acetate extract	HCT-116 and SW-620	0.5 and 1.0 mg/L	Suppressed the clone formation, invasion and migration of CRC by suppressing the PI3K/Akt pathway	Li Y et al. (2022)
			AOM/DSS Mode	5, 10 or 20 mg/kg		
	Asparagus (ASP)	Water extract	HCT116, LOVO, and LO2	50, 100, 200 µm/mL	Inhibited proliferation, invasion and migration of HCT116 and LOVO cells via PI3K/AKT/mTOR	Liang et al. (2022)
	Scutellaria barbata D.Don (SB)	Ethanol extract	HCT-8	0.125, 0.25, 0.5 mg/mL	Inhibited the migration and invasion ability of HCT-8 cells via PI3K/Akt and TGF-β pathways	Jin et al. (2017)
	Sanguisorba officinalis L. (DY)	Aqueous extract	RKO-P/R and HCT15-P/R cells	124.2 and 105.2 μg/mL	Suppressed the growth and metastasis of 5-FU-sensitive and -resistant CRC via inhibition of the Wnt pathway	Yi et al. (2022)
	Angelica sinensis	Aqueous extract	HCT116 cells	36.2 mg/kg, 72.4 mg/kg	Inhibited the viability, metastasis, and invasion of HCT116 cells, especially under the influence of TAMs	Hao et al. (2022)
Other mechanisms	Sanguisorba officinalis L. (DY)	Water extract	RKOP, HCT15P, RKOR and HCT15R	100 µg/mL	Increased the susceptibility of 5-FU to drug-resistant CRC cells via the Ras/MEK/ERK and PI3K/Akt pathways	Zhang et al. (2023)
	Portulaca oleracea extract (POE)	Water extract	AOM/DSS method	200, 800 mg/kg	Downregulated c-Myc and cyclin D1 expression, reduced gut microbiota imbalance through inhibiting the Wnt/β-catenin signaling pathway	Yi et al. (2022)
	Curcumae longae Rhizoma	Aqueous extract	SW480/5-FuR	10 mg/mL	Reversed 5-Fu resistance in CRC by inactivating TLR4/PI3K/AKT/mTORC1 pathway	Teng et al. (2022)
	Juniperus communis (JCo)	Aqueous extract	HT-29, ATCCs HTB-38	10 mg/mL	Induced cell cycle arrest at the G0/G1 phase via regulation of p53/p21 and CDK4/cyclin D1 and induced cell apoptosis	Lai et al. (2021)

### 3.3 Other mechanisms

As we all known, 5-fluorouracil (5-FU) was the first-line cure of medicine in treatment CRC. But, the acquisition of chemotherapy drug resistance always caused of cancer treatment failure. Sanguisorba officinalis L. (DY) increased the susceptibility of 5-FU to drug-resistant CRC cells via the Ras/MEK/ERK and PI3K/Akt pathways (Zhang et al., 2023). Coupled with Sanguisorba officinalis L., Portulaca oleracea extract (POE) downregulate c-Myc and cyclin D1 expression, reducing gut microbiota imbalance through inhibiting the Wnt/β-catenin signaling pathway (Yi et al., 2022). What’s more, Curcumae longae Rhizoma can reverse CRC 5-Fu resistance by inactivating the TLR4/PI3K/AKT/mTORC1 pathway. Curcumae longae Rhizoma combined with 5-Fu can induce cell apoptosis by inhibiting bcl-2 and activating caspase-3 and Bax, thereby reversing 5-FU resistance (Teng et al., 2022). Additionally, Juniperus communis (JCo) is a well-known plant to treat cancer in traditional herbal medicine. The results showed that JCo, which was extracted by steam distillation, had a synergistic effect with 5-FU in CRC cells. In fact, the cell cycle played an important role in treating CRC. Jco extract can reduced cell cycle arrest to inhibit CRC growth (Lai et al., 2021).

## 4 Application of components in CRC

Compared with classical Chinese medicine prescriptions and the previous clinically applied herbs for CRC, TMC components have the benefit of being single, administered in small dosages, presenting clear effectiveness indicators, and a precisely defined mechanism of action (Guo T-H et al., 2022). According to their chemical structure, components comprise alkaloids, flavone, glycosides, and other components. Extensive research has made considerable progress in exploring the properties of components for treatment CRC (Guo T-H et al., 2022). Their mechanism of action has become more apparent, promoting the precise treatment of CRC by components. Details of the anti-CRC activity of the TMC components are shown in Table 3.

**TABLE 3:** Lists of TCM Identified compounds with potential anti-CAC action.

Regulatory mechanism	Compounds	Model	Dosage	Effects and potential mechanism	Ref
Inducing apoptosis and inhibiting proliferation	Berberine	HCT116 SW480	2.5–120 μg/mL	Inhibited the malignant phenotypes of CRC through diminishing Hedgehog signaling cascade.	Sun et al. (2023)
	Lycorine	SW480 and RKO	10 µM, 20 µM, 30 µM, 40 µM	Induced the activation of the caspase-dependent mitochondrial apoptotic pathway	Wu et al. (2018)
	Coptisine	HCT-116	7.03 µM, 14.05 µM, 28.11 µM	Induced apoptosis in HCT116 cells through PI3K/Akt and mitochondrial-assiciated apoptotic pathway	Han et al. (2018)
	Homoharringtonine	LoVo, Caco-2 and SW480	0.32 µM, 0.56 µM, 0.38 µM	Suppressed LoVo cell growth *in vitro* and *in vivo*, and induced apoptosis and cell cycle arrest at the S phase	Shi et al. (2020)
	Evodiamine	SW480	100 and 200 µM	Binded to the ordered domain (α-helix) of NF-kB to achieve its anti-inflammatory and antitumor effects	Zhang Y et al. (2022)
		C57BL/6 mice	10 mg/kg,30 mg/kg		
		(Apc)MinC/Gpt C57BL/6 mice	10 mg/kg		
	Scutellarin	AOM/DSS C57BL/6 mice	25, 50, 100 mg/kg	Ameliorated AOM/DSS-caused CAC in mice and induced apoptosis in CAC tissues of mice, by inhibiting NF-κB and Hedgehog signaling axis	Zeng et al. (2022)
	Celastrol	HCT116, SW480	1.25, 2.5, 5 µm	Downregulated Nur77, induced apoptosis and inhibited proliferation in CRC cells	Zhang W et al. (2022)
		BALB/c nude mice	1.25, 2.5 mg/kg		
	Genistein	HT29, SW620	1, 5, 50, 100 µM	Decreased cell viability and produced G2/M arrest, increased H2O2, and produced filopodia in SW620 cells	Alorda-Clara et al., 2022
	Matrine	NCM460, HCT116 and SW480	1.2, 2.4, and 3.6 μM	Triggered apoptosis of HT29 and DLD1 by suppressing the miR-10b-5p/PTEN pathway	Cheng et al. (2022)
	Ginsenoside Rh2	HCT116, SW620	10 µM, 20 µM	Alleviated the accelerating effect on Wnt pathway activity, cell proliferation/migration, and colony formation	Li S et al. (2023)
	Fisetin	SW480	30 µM	Induced apoptosis in colorectal cancer cells by suppressing autophagy and downregulated nuclear factor erythroid 2-related factor 2 (Nrf2)	Pandey and Trigun (2023)
	Hesperetin	Wistar	25 mg/kg	Suppressed of oxidative stress and reducted in cell proliferation and the enhancement of apoptosis	Hassan et al. (2023)
	Curcumin	HCT-116, SW620	10 µM, 20 µM, 40 µM	Regulated the CDCA3/CDK1 pathway, thereby inhibited proliferation in colorectal cancer	Liu et al. (2023)
		BALB/c nude mice	200 mg/kg		
	Dendrobium polysaccharides	Zebrafish Xenograft Model	250 µg/mL	Induced apoptosis in human colorectal cancer	Tao et al. (2021)
	Astragalus polysaccharides				
	Shiitake mushroom polysaccharides				
	Apigenin	LS-174T, HCT-8, HT-29, HCT-116	40 µM	Restricted the glycolysis of LS-174T and HCT-8 cells by targeting the K433 site of PKM2	Shi et al. (2023)
	Oridonin	RKO, LOVO	20, 25, 30 µM	Upregulated TP53, inhibited TCF4 transactivation via inhibiting the TP53/TCF4 axis	Zhou et al. (2023)
Inhibiting migration, invasion and adhesion	Ginsenoside Rg3	HUVEC	25, 50 µM	Suppressed the loop formation and migration of human umbilical vein endothelial cell (HUVEC)	Nakhjavani et al. (2021)
	Polysaccharide (EPS1-1)	CT26	0.1, 0.2,0.4 mg/mL	Inhibited the expression levels of matrix metalloproteinases (MMPs), vascular endothelial growth factor (VEGF) and microvessel density (MVD)	Yu et al. (2018)
	Quercetin	Wistar	50 mg/kg	Inhibited lipid and protein peroxidation by modulating the activity of the Nrf2/keap1	Darband et al., 2020
	Atractylenolide I	HCT116	25, 50, 100, 200 µM	Increased oxaliplatin sensitivity via the PDK1/FoxO1 axis and inhibited the proliferative, migratory and invasive abilities	Sun et al. (2022)
	8-gingerol	HCT116, DLD1	70, 100 µM	Decreased in migration and invasion of CRC by targeting the EGFR/STAT/ERK pathway	Hu et al. (2020)
	Andrographolide	HCT116	5, 10, 20 µM	Exhibited significant colorectal cancer activity by inhibiting the Src/MAPKs/AP-1 signaling pathways	Yuan et al. (2018)
	Sophoridine	HCT116, RKO, SW480	80 µM, 160 µM	Inhibited growth and invasion in colorectal cancers by MAPKAPK2	Wang et al. (2019)
	Rg1	C57BL/6 mice	50, 100, 150 μM	Inhibited the lung metastasis of CRC	Liu et al. (2024)
	liquiritigenin	HCT116	10, 20, 50, 100 µg/mL	Downregulated the expression of Runx2 and inhibited PI3K/AKT to inhibit the invasion and EMT	Meng and Lin (2019)
	Berberine	SW620, HCT116 and LOVO	50, 100, 150 µM	Inhibit mesenchymal epithelial transformation (MET) via reducing HEY2, E-cadherin, β-catenin and cyclin D1	Ni et al. (2022)
Other mechanisms	D3-3	HCT116	5, 10, 20 µM	Promoted CRC cells to release the ferrous ion in autophagy-dependent manner	Zhu et al. (2024)
	Tangeretin Synergizes	HCT-116	0.41 µM	Induced autophagy through MicroRNA-21 in colorectal cancer cells	Bai et al. (2022)
	Quercetin	HCT116, SW480	20, 40, 80, 120 µM	Regulated autophagy, and enhance the sensitivity of CRC for 5-FU via Drp-1-mediated mitochondrial fragmentation	Li et al. (2024)
	kaempferol	HCT8-R	50 µM, 100 µM	Overcame resistance to 5-Fu therapy by regulating the miR-326-hnRNPA1/A2/PTBP1-PKM2 axis	Wu et al. (2022)
	Solanine	HCT116 and SW480	20 µM	Regulated the ALOX12B/ADCY4 molecular axis to induce typical ferroptotic changes in CRC cells	Ma et al. (2024)
	GRh3	HT29, HCT116	20 µM, 40 µM, 80 µM	Triggered pyroptotic cell death and ferroptotic cell death in CRC cells through the Stat3/p53/NRF2 axis	Wu et al. (2023)
	Luteolin	HT-29	50, 100, 150 µM	Inhibits the proliferation of colon cancer cells through the pyroptosis pathway	Chen et al. (2022)
	Ginsenoside R1	AOM/DSS C57BL/6 mice	None	Reduced the levels of TNF-α, IL-6, IL-17A, IL-33, IL-1β, and IL-22, increased the level of IL-10, and also changed the gut microbiota composition.	Wang et al. (2023)
	Baicalin	HCT116 CT26	0,5,10,20uM	Triggered apoptosis, inhibited migration, and enhanced anti-tumor immunity in colorectal cancer via TLR4/NF-κB signaling pathway	Yang et al., 2020
		Balb/c mice	20 mg/kg, 40 mg/kg		
	Emodin	SW620 and HCT116	40 µM	Decreased in inflammatory cell infiltration and pro-inflammatory enzyme expression in the tumour microenvironment	Zhang N et al. (2021)
		AOM/DSS C57BL/6 mice	50 mg/kg		

### 4.1 Inducing apoptosis and inhibiting proliferation

Sun et al. found that berberine induced apoptosis and blocked the cell cycle at phase G0/G1 in HCT116 and SW480 with a dampened hedgehog pathway (Sun et al., 2023). As showed by the increase of the ratio of Bax/Bcl-2 and mitochondrial depolarization, Lycorine induced mitochondrial apoptosis by targeting the STAT3 pathway (Wu et al., 2018). Besides, Coptisine also activated mitochondrial apoptosis of HCT-116 by down-regulating pro-caspase 3, Bcl-2 and upregulating Bax, cytochrome c and cleaved caspase-3 expression (Han et al., 2018). Homoharringtonine regulated cyclinA2 and CDC2 in the Bcl-2 apoptosis pathway by inhibiting the PI3K/AKT pathway of Lovo cells. The study showed that Homoharringtonine significantly suppressed LoVo cell growth *in vitro* and *in vivo* (Shi et al., 2020).

Evodiamine inhibited the NF-κB pathway by binding to the α-helix of NF-κB, inhibiting colon cancer proliferation (Zhang Y et al., 2022). Scutellarin significantly ameiorated tissue apoptosis in the AOM/dss mouse model by inhibiting NF-κB and Hedgehog signaling axis (Zeng et al., 2022). Celastrol can also regulate the NF-kB/COX-2 signaling pathway, activate cysteine-dependent apoptosis, and promote G1 cell cycle arrest, thereby inhibiting the proliferation and inducing apoptosis of CRC (Zhang H et al., 2022). Genistein could effectively decrease the viability of HT29 and SW620 cells and found that intracellular NF-KB was translocated from the cytoplasm to the nucleus, which proved that genistein could decrease cell viability of colon cancer cells and inhibit the proliferation by increasing the oxidative stress and inflammatory response of colon cancer cells (Alorda-Clara et al., 2022).

Compared to inhibiting the NF-κB pathway, Chen et al. discovered that Matrine triggered apoptosis of HT29 and DLD1 by suppressing the miR-10b-5p/PTEN pathway (Cheng et al., 2022). Ginsenoside Rh2 inhibited Wnt pathway activity and inhibits cell proliferation/migration and colony formation (Li S et al., 2023). Besides, Fisetin induces apoptosis by down-regulating nuclear factor erythroid 2-related factor 2 (Nrf2) in CRC (Pandey and Trigun, 2023). In the same animal model, the study found that Hesperetin reduced the occurrence of CRC induced by 1,2-dimethylhydrazine in Wistar rats by inhibiting oxidative stress, enhancing antioxidant, anti-inflammatory and apoptosis effects (Hassan et al., 2023). Liu F et al. reported that the administration of curcumin significantly suppressed the size of xenograft tumors. Mechanistic exploration determined that curcumin can target miR-134-5p expression and regulate the CDCA3/CDK1 pathway, thereby inhibiting proliferation in CRC (Liu et al., 2023). Additionally, Other authors have established a zebrafish transplantation model and demonstrated that dendrobium polysaccharides, astragalus polysaccharides, and shiitake mushroom polysaccharides can effectively inhibit the growth of HT29 cells. Their mechanism of action may involve immune modulation and the induction of apoptosis in tumor cells (Tao et al., 2021).

Besides, Apigenin was positively correlated with pyruvate kinase M2 (PKM2) expression in LS-174T cells and HCT-8 cells. The characterized of Apigenin suppressed cell proliferation and increased of apoptotic effects (Shi et al., 2023). What’s more, Oridonin, a diterpenoid compound extracted from Rabdosia rubescens, has been indicated to inhibit the proliferation of CRC. Oridonin promoted CRC cell death, upregulating TP53, inhibiting TCF4 transactivation via inhibiting the TP53/TCF4 axis (Zhou et al., 2023).

### 4.2 Inhibiting migration, invasion and adhesion

Metastasis of colorectal cancer is a complex pathophysiological process that involves multiple factors and steps. One crucial factor is angiogenesis, which is necessary for primary tumour metastasis and is regulated by both pro-angiogenic and anti-angiogenic factors. Ginsenoside Rg3 (Rg3) has stereoselective activities to decrease the expression of vascular endothelial growth factor receptor 2(VEGFR2) and aquaporin1. Through response surface methodology, Rg3 can significantly suppress the loop formation and migration of human umbilical vein endothelial cell (HUVEC) (Nakhjavani et al., 2021). Polysaccharide (EPS1-1) dose-dependently suppressed the migration, invasion and adhesion abilities of CT26 cells. EPS1-1 dramatically inhibited the expression levels of matrix metalloproteinases (MMPs), vascular endothelial growth factor (VEGF) and microvessel density (MVD) in CT26 cells (Yu et al., 2018).

The components of TCM can effectively exert the inhibition of migration of CRC via multi-pathway. Quercetin can effectively suppress the migration and invasion of RKO cells through modulation of the JNK pathway (Trinh et al., 2022). Besides, Atractylenolide can affect PDK1/FoxO1, AKT/mTOR, and JAK/STAT3 pathways, inhibiting cancer cells’ proliferation, migration, and invasive ability (Li et al., 2020; Wang K et al., 2020; Sun et al., 2022). 8-gingerol, which is extracted from ginger, resulted in dose-dependent decrease in migration and invasion of CRC by targeting the EGFR/STAT/ERK pathway (Hu et al., 2020). Andrographolide has exhibited significant colorectal cancer activity by inhibiting the Src/MAPKs/AP-1 signaling pathways in a concentration-dependent manner (Yuan et al., 2018). Through cell heat shift experiments and drug affinity response target stability experiments, MAPK/APK2 plays a crucial role in Sophoridine inhibiting the growth and invasion of HCT116, SW480, and RKO(Wang et al., 2019). Bufalin, as the main active monomer of huachanshu, induced M2-type polarization and inhibited CRC metastasis via the SRC-3/IL-6 pathway (Tang et al., 2024). Rg1 also inhibited migration of CRC. Liu et al. found that the combination of rosmarinic acid (RA) and Rg1 can have anti-metastatic effects against CRC in regulating of PD-1/PD-L1 in CRC. Thus, Rg1 can inhibit the lung metastasis of CRC (Liu et al., 2024).

Besides, EMT was related to invasion and metastasis of tumor cells via inducing loss of cell-cell junctions and apicobasolateral polarity (Zhang N et al., 2021). Meng et al. found that liquiritigenin, a flavonoid extracted from the roots of Glycyrrhiza uralensis Fisch, downregulated the expression of Runx2 and inhibited PI3K/AKT to inhibit the invasion and EMT in HCT116 cell (Meng and Lin, 2019). What’s more, berberine treatment can inhibit mesenchymal epithelial transformation (MET) via reducing HEY2, E-cadherin, β-catenin and cyclin D1 (Ni et al., 2022). Besides, peroxisome proliferator-activated receptor gamma coactivator 1α (PGC-1α), being a regulator of mitochondrial function, can promote ABCA1 expression to promote CRC metastasis through EMT. Chen et al. found that the natural compound Isoliquiritigenin (ISL), as an inhibitor of PGC-1α, targeted ABCA1 and reduced CRC metastasis by inhibiting EMT (W. Chen W et al., 2023). All in all, the components of TCM can effectively regulate multiple factors and steps of CRC to inhibiting migration, invasion and adhesion.

### 4.3 Other mechanisms

There are some different mechanisms, such as autophagy and ferroptosis, being implicated in the cell death of cancer cells (Gao et al., 2022). D3-3 stemming from sinomenine, is a new compound through synthesis and design. D3-3 apparently promote CRC cells to release the ferrous ion in autophagy-dependent manner (Zhu et al., 2024). On the other hand, Tangeretin regulated miRNA-21 to induce autophagy by synergizing with 5-Fluorouracil in CRC (Bai et al., 2022). As the same mechanism, quercetin also could regulate autophagy, and enhance the sensitivity of CRC for 5-FU via Drp-1-mediated mitochondrial fragmentation (Li et al., 2024). Kaempferol regulated the miR-326-hnRNPA1/A2/PTBP1-PKM2 axis to overcome resistance to 5-Fu therapy (Wu et al., 2022). Solanine regulated the ALOX12B/ADCY4 molecular axis to induce typical ferropto in CRC cells. Simutaneously, solanine-induced ferroptosis is suppressed by silencing ALOX12B (Ma et al., 2024). Ginsenoside Rh3 triggered pyroptotic and ferroptotic cell death in CRC cells through the Stat3/p53/NRF2 axis while causing minimal damage to normal cells. These findings demonstrate remarkable anticancer potential for GRh3 (Wu et al., 2023). Luteolin experiments confirmed that it inhibits the proliferation of colon cancer cells through the pyroptosis pathway. Luteolin treatment increased the expression of Caspase1 and Gasdermin D. And we observed through immunofluorescence co-localization that NLRP3/Gasdermin D combined and inhibited CRC (Chen et al., 2022).

Additionally, the component of TCM was related to the tumor microenvironment. Ginsenoside R1 significantly decreased intestinal inflammatory factors TNF-a, IL-6, IL-1β, and IL-22. It also altered the composition of gut microbiota, effectively alleviating chronic inflammation and repairing the intestinal microenvironment in the AOM/DSS model (Wang et al., 2023). Baicalin could prompt apoptosis in both HCT116 and CT-26 by activating the TLR4/NF-kB pathway, significantly reducing the proliferation of colon cancer cells. Alongside this, baicalin could improve the anti-tumor immune function, down-regulating PD-L1 expression and upregulating the CD4^+^ and CD8^+^ T cell ratio, thereby improving the tumor immune microenvironmen (Yang et al., 2020). What’s more, it has been reported that using Emodin on the AOM/DSS mouse model decreased inflammatory cell infiltration and pro-inflammatory enzyme expression in the tumor microenvironment while increasing CD3 (+) T-lymphocyte levels. Moreover, it effectively reduced the cell viability of SW620 and HCT116 cells in in vitro experiments (Zhang Y et al., 2021).

## 5 Discussion

Colorectal cancer represents a significant global health burden, with high morbidity and mortality rates (Siegel et al., 2023). It is frequently diagnosed and approximately 35% of patients are found to have intermediate to advanced stage cancer at initial diagnosis. According to clinical practice guidelines developed by the National Comprehensive Cancer Network and the European Society for Medical Oncology, adjuvant chemotherapy with the FoLFox regimen is the standard of care for patients with intermediate to advanced colorectal cancer. This regimen has also been demonstrated to significantly enhance patient prognosis and increase overall survival (Guo et al., 2016). However, chemotherapy also has cytotoxic effects and is prone to causing adverse reactions, such as the inhibition of bone marrow haematopoiesis, digestive dysfunction, hand-foot syndrome, and even life-threatening conditions (Guo et al., 2016). TCM anti-tumour treatment options have been proposed by researchers as a response to these adverse effects.

TCM has a distinct theoretical framework with holism and dialectics at its core. It is a medical science developed through the practical experiences of Chinese people from all ethnic backgrounds in treating various diseases and has gained extensive clinical knowledge. TCM have focused on reducing adverse reactions and preventing tumor recurrence and metastasis. Research has shown that TCM can lower the tumor recurrence and metastasis rate in patients with CRC, as well as reduce the occurrence of complications.

This paper presents a detailed analysis of prescriptions, herbs, and components. The study and implementation of prescriptions in TCM demonstrate its distinctive holistic approach to therapy, characterized by applying multi-component and multi-target strategies. Similar to the compound presented in this paper, it is categorized based on its primary effects, including inhibition of apoptosis and proliferation, inhibition of metastasis, the regulation of gut microbiota and other mechanisms (Table 1).

The use of TCM in the treatment of CRC is becoming increasingly widespread. It is often used in conjunction with conventional Western medicine or as a standalone treatment. Despite the considerable progress made in TCM research on CRC, with a wide range of research topics and directions, there are still some outstanding issues. These mainly include: first and foremost, in the research of TCM against CRC, most studies focus on herb or compound of TCM, with fewer studies on TCM prescriptions. In reality, TCM prescriptions have multiple targets and roles. For example, Gegen Qinlian Decoction can block PD-1 by reshaping the gut microbiota and tumor microenvironment in CRC (Lv et al., 2019). Meanwhile, Gegen Qinlian decoction can increase the activity of Nrf2/ARE signaling and enhance the effect of antioxidant stress (Lin et al., 2022). Second, the observation indexes are relatively broad in clarifying Chinese medicine’s clinical treatment of CRC. The study of TCM in the treatment of CRC lacks precise observation indexes, which undermines its ability to convincingly elucidate therapeutic efficacy. Third, Currently, the multi-component and multi-target nature has also limited research related to TCM prescriptions. Clarifying the material basis, targets, and molecular biological mechanisms is challenging. Finally, The TCM theory emphasizes a holistic approach (Chen J-F et al., 2023). An identified compound represents only one constituent among the many ingredients found in TCM prescriptions. The diverse biological impacts resulting from the interdependence of the numerous ingredients in TCM still need to be fully comprehended. Hence, there is a pressing requirement for further excavation techniques and methods to investigate TCM and uncover its role in treating colorectal cancer and its mechanism of action. This paper covers a comprehensive analysis of the research advancements made in TCM prescriptions, herbs, and components, offering a specific theoretical basis for researchers exploring the treatment of CRC with TCM.

## 6 Summary

TCM is often utilised for anti-tumour purposes and has showcased encouraging anti-tumour efficacy in research studies. As science and technology progress, there is an expectation that research on the anticancer mechanism of traditional Chinese medicine will advance and improve. Cutting-edge medical research technology enables researchers to identify disease targets and apply multi-component, multi-pathway, and multi-target treatment of TCM to treat CRC. This approach is also an important avenue for studying TCM treatment of CRC in the future. Currently, there are still some shortcomings in the research of TCM for the treatment of CRC. However, it is believed that with the continued development of medical science and technology, the field of Chinese medicine’s anti-tumour properties will deepen, leading to more abundant results in the research of colorectal cancer.

## Abbreviations

CRC, Colorectal cancer; TCM, Traditional Chinese medicines; VEGF, endothelial growth factor; QFGs, Qingjie Fuzheng granules; SJYLF, Sanjie Yiliu formula; SJZD, Sijunzi Decoction; WTX, Wei-Tong-Xin; YYFZBJS, Yi-Yi-Fu-Zi-Bai-Jiang-San; XYS, Xiaoyaosan; JPJDR, JianPi JieDu Recipe; AAM, Astragalus Atractylodes mixture; GQ, Gegen Qinlian decoction; SYD, Shaoyao decoction; HXZQ, Huoxiang Zhengqi; XJR, Xiaoai Jiedu recipe; PZH, Pien-Tze-Huang; WMW, Wu Mei Wan.
